# Baby Boomers as Caregivers: Results From the Behavioral Risk Factor Surveillance System in 44 States, the District of Columbia, and Puerto Rico, 2015–2017

**DOI:** 10.5888/pcd17.200010

**Published:** 2020-08-13

**Authors:** Christina E. Miyawaki, Erin D. Bouldin, Christopher A. Taylor, Lisa C. McGuire

**Affiliations:** 1 Graduate College of Social Work, University of Houston, Houston, Texas; 2 Alzheimer’s Disease and Healthy Aging Program, Centers for Disease Control and Prevention, Atlanta, Georgia; 3 Department of Health and Exercise Science, Appalachian State University, Boone, North Carolina

## Abstract

**Introduction:**

Baby boomers, people born from 1946 through 1964, represent a substantial portion of the US population. Generally, baby boomers have more chronic disease and disability than those in the previous generation. Frequently, they also provide informal care to others. The objective of our study was to estimate the prevalence of informal caregiving among baby boomers and compare the health of baby boomer caregivers and noncaregivers.

**Methods:**

Using data from the Behavioral Risk Factor Surveillance System (2015–2017) for 44 states, the District of Columbia, and Puerto Rico, we classified 109,268 baby boomers as caregivers or noncaregivers and compared their general health (poor or fair vs good, very good, or excellent), chronic health conditions, and frequent mental distress (FMD). FMD was defined as 14 days or more of poor mental health in the past month. We used log-binomial regression to calculate prevalence ratios, adjusted for age and sex (aPRs), and to separately estimate aPRs for fair or poor health and FMD or at least one chronic health condition.

**Results:**

One in 4 baby boomers (24.2%) were caregivers. In adjusted models, male caregivers had a higher prevalence of fair to poor health than noncaregivers (aPR = 1.17; 95% confidence interval [CI], 1.06–1.29; *P* = .001). More caregivers than noncaregivers had at least 1 chronic health condition (aPR = 1.10, 95% CI, 1.07–1.13; *P* < .001) and more often had FMD (aPR = 1.39; 95% CI, 1.26–1.53; *P* < .001).

**Conclusion:**

Our study showed these caregivers had more chronic health conditions and more often had FMD than noncaregivers. The health of baby boomer caregivers is a public health priority, as these caregivers might need support to maintain their own physical and mental health.

**Summary**

**What is already known on this topic?**

In the United States, baby boomers provide much informal care for people with health problems and disabilities. Men in these caregiving roles more often report poor general health than women caregivers from the same generation.

**What is added by this report?**

Caregivers who are baby boomers more commonly report frequent mental distress and have more chronic health conditions than noncaregivers, which might put them at risk of becoming care recipients.

**What are the implications for public health practice?**

For caregivers to maintain their health and continue providing care, efforts must be made to reduce the negative health effects of caregiving and provide support to caregivers for managing stress and chronic health conditions.

## Introduction

In the United States, much of the care for people with health problems, long-term illness, or disability is provided by family members or friends in the community (1). In 2015, 43.5 million adults were providing this informal care (2). Baby boomers, born from 1946 through 1964 and currently in middle to older age, have more chronic disease, more disability, and lower self-rated health than those of the previous generation (3), and they might also provide substantial care for others. This care might be for a partner or friend of a similar age with a chronic condition, long-term illness, or disability (4), an older parent who might be declining cognitively, or a family member with a health condition, injury, or disability (5).

Although providing informal care can bring many benefits, it is also a source of a chronic stress (6). Caregivers might experience this stress because of the physical demands of caregiving, the challenges of balancing work and other responsibilities with the caregiving role, the trouble with managing problematic behaviors of the people they care for, or the emotional difficulty of watching a loved one’s health decline (6,7). Stress coping models or process models have shown that the strain associated with caregiving can result in psychological distress and interference with the immune system (8) and cardiovascular functions (6,7). Caregivers might also engage in health behaviors that contribute to negative health outcomes because of limited time to be physically active, attend medical appointments, or manage their own chronic conditions. Together, these physiological and behavioral changes increase the likelihood of developing new physical and mental health conditions (6,7).

By providing care to others, baby boomer caregivers who might have their own physical or mental challenges could be in a position that negatively affects their own health (6,7,9). Despite this supposition, few studies exist on caregiving among baby boomers and the self-reported health of this population. Therefore, it is imperative that we learn about current health conditions and caregiving situations of baby boomers so that we can prevent declines in their health to the extent possible, especially given the anticipated shortage of available caregivers for this generation (10). The objectives of our study were to estimate the prevalence of informal caregiving among baby boomers, to describe the type of care they provide, and to evaluate whether their overall health and mental health differ from their noncaregiving peers.

## Methods

### Data source

We used 3 years of data (2015–2017) from the Behavioral Risk Factor Surveillance System (BRFSS). BRFSS is a state-based landline and cellular telephone survey of noninstitutionalized, community-dwelling adults aged 18 years or older, conducted by state health departments with support from the Centers for Disease Control and Prevention (CDC) (www.cdc.gov/brfss). The Caregiver Module is an optional set of 9 questions developed for the BRFSS that states may choose to administer. For 2015–2017, 44 states, the District of Columbia, and Puerto Rico administered these caregiving-related questions. If a state included the module in more than 1 year, we included only the most recent data in the analysis. Because BRFSS does not provide birthdates, we considered baby boomers (born from 1946 through 1964) by their age in years at the time of survey (ie; 50–69 in 2015, 51–70 in 2016, 52–71 in 2017). Our study was reviewed by Appalachian State University and classified as exempt.

### Caregiver status

We classified respondents as caregivers if they answered yes to the following Caregiver Module screening question, “People may provide regular care or assistance to a friend or family member who has a health problem, long-term illness, or disability. During the past month, did you provide any such care or assistance to a friend or family member?” We classified respondents as noncaregivers who answered no to the caregiver screening question.

### Health status

Respondents rated their general health as excellent, very good, good, fair or poor, and we classified them as fair or poor versus excellent, very good, or good. We evaluated the presence of the following chronic health conditions: arthritis, current asthma, cardiovascular disease (angina, stroke, or myocardial infarction), diabetes (excluding gestational or prediabetes), cancer other than skin cancer, and chronic obstructive pulmonary disease. We created a dichotomous variable to indicate whether respondents had at least 1 of these chronic health conditions and required responses to at least 4 of the 6 items for classification as having a chronic health condition or not. Frequent mental distress (FMD) was determined by answering this question, “Now, thinking about your mental health, which includes stress, depression, and problems with emotions, for how many days during the past 30 days was your mental health not good?” Consistent with previous research and recommendations from CDC (11), we classified respondents as having FMD if they reported 14 days or more of poor mental health in the past 30 days.

### Covariates

All data collected through the BRFSS are self-reported. We included data on respondents’ sex and age in years. We created variables with the following categories for descriptive purposes: age group (50–54, 55–59, 60–64, 65–71, using the imputed age variable [0.3% of respondents were missing self-reported age]), race/ethnicity (non-Hispanic black, non-Hispanic white, non-Hispanic Asian or Pacific Islander, Hispanic any race, non-Hispanic other race or multiracial), highest level of educational attainment (less than high school, high school or equivalent, some college, college graduate or higher), and employment status (employed or self-employed, out of work, homemaker, student, retired, unable to work).

Among caregivers, we categorized the care recipient’s relationship to the caregiver as parent, spouse or partner, other family member, or nonfamily member. We created dichotomous variables to indicate caregiving duration (<2 years, ≥2 years), caregiving hours (<20 hours per week or ≥20 hours), and caregiving tasks (personal care and household tasks). Household tasks included activities such as cleaning, managing finances, and preparing meals and personal care involving more hands-on care such as dressing, bathing, and feeding. If the unweighted denominator was less than 50 or the relative standard error (calculated as weighted standard error divided by weighted percentage, multiplied by 100) was greater than 30, we did not report the estimate because they may be unstable.

### Statistical analysis

We included BRFSS respondents who were classified as baby boomers and had no missing values for our primary variables: caregiving status, general health, FMD, and at least 1 chronic health condition. We also limited our sample to respondents who had a valid (dichotomous choice) response for sex, because we included this as a covariate in our regression models. Among 111,672 baby boomers who had a response recorded for the caregiving screening question, we excluded 2,404 (2.2% unweighted) because of missing information; therefore, our final sample size was 109,268.

We calculated the weighted proportion of caregivers overall. We also calculated weighted proportions to describe the demographic and health status characteristics of baby boomer caregivers and noncaregivers and used χ^2^ tests to compare proportions across groups. Finally, we examined caregiving characteristics, such as total caregiving duration, weekly caregiving hours, and caregiving tasks. We used separate log-binomial regression models to estimate the adjusted prevalence ratios (aPRs) for having fair or poor health, FMD or at least one chronic health condition, adjusting for age in years and sex. We considered terms for both age and age^2^ in our models. The primary model was not adjusted for sociodemographic characteristics because the focus of our study was on the overall association between caregiving status and health outcomes. However, we conducted a sensitivity analysis adjusting for education category and race/ethnicity to represent socioeconomic position. Because of the lack of diversity in race/ethnicity among respondents, we could only adjust for non-Hispanic white race/ethnicity versus all other groups. We tested each model for effect modification by sex, including an interaction term between caregiver status and sex. We established *P* < .05 to indicate significance, including effect modification.

Data were weighted using the appropriate weight variable in the BRFSS public data file, based on the survey version(s) of the Caregiver Module that appeared in each state (12). Primary sampling units and stratum weights were also included in our weighting statements to appropriately calculate standard errors. All analyses were conducted using survey (SVY) commands (StataCorp, LLC) with subpopulation statements as appropriate (eg, restricting to respondents who were baby boomers with no missing covariates) in Stata version 13.1.

## Results

Of the 109,268 baby boomers surveyed, 24.2% were caregivers, and of all caregivers, 38.5% were baby boomers. Caregivers were slightly younger (mean [SD] = 59.3 [6.6] y) than noncaregivers (mean [SD] = 59.8 [6.6] y) (*P* < .001) and were more often women, non-Hispanic white, and college-educated (Table 1). Employment status was similar among caregivers and noncaregivers; about half of the baby boomers were employed, and about 25% were retired.

**Table 1:** Characteristics of Baby Boomer Caregivers and Noncaregivers, Behavioral Risk Factor Surveillance System, 2015–2017^a^

Characteristic	Caregivers, Weighted % (Unweighted n = 26,617)	Noncaregivers, Weighted % (Unweighted n = 82,651)	*P* Value^b^
**Sex**			
Female	61.7	49.0	<.001
**Age group, y**			
50–54	26.7	23.5	<.001
55–59	25.9	25.5	
60–64	26.0	26.1	
65–71	21.3	24.9	
**Race/ethnicity**			
Non-Hispanic black	11.2	10.5	.25
Non-Hispanic white	74.0	68.8	<.001
Hispanic	8.6	12.6	<.001
Non-Hispanic Asian or Pacific Islander	1.1	4.1	<.001
Other race or multiracial	3.5	2.5	.001
Missing	1.5	1.6	NA
**Education**			
Less than high school	9.0	13.5	<.001
High school degree or equivalent	27.9	27.9	
Some college	35.8	29.7	
College graduate	27.1	28.7	
Missing	0.2	0.2	NA
**Employment status**			
Employed or self-employed	51.7	52.3	.15
Unemployed	5.4	4.8	.11
Homemaker	6.2	5.2	.07
Student	NA	0.2	NA
Retired	24.9	25.7	0.35
Unable to work	11.7	11.4	0.51
Missing	0.9	0.5	NA
**General health status**			
Excellent, very good, or good	75.8	77.2	.09
Fair or poor	24.2	22.8	
**Chronic health conditions diagnosed**			
Arthritis	44.4	37.0	<.001
Asthma (current only)	10.9	9.1	.002
Cancer (except skin)	10.1	9.5	.36
Cardiovascular disease^c^	13.0	12.3	.36
Diabetes (except gestational)	17.2	18.2	.17
COPD	11.2	9.2	<.001
**≥1 Chronic health condition diagnosed**	63.4	57.3	<.001
**Frequent mental distress (≥14 days of poor mental health in the past 30 days)**	15.2	10.3	<.001

Abbreviations: COPD, chronic obstructive pulmonary disease; NA, not applicable or not reported because relative standard error >30.0, indicating unstable estimate.

a 2015 states: Alabama, Florida, Idaho, Illinois, Indiana, Iowa, Kentucky, Louisiana, Maine, Mississippi, Nebraska, Pennsylvania, South Carolina, Virginia, West Virginia, Wisconsin, Wyoming; 2016 states/territories: Arizona, Arkansas, California, Colorado, Connecticut, District of Columbia, Georgia, Minnesota, Missouri, Montana, Nevada, North Dakota, Ohio, Puerto Rico, South Dakota, Tennessee, Texas; 2017 states: Alaska, Hawaii, Kansas, Maryland, Michigan, New Jersey, New Mexico, New York, Oklahoma, Oregon, Rhode Island, Utah.

b *P* value based on χ^2^ test of weighted proportions.

c Cardiovascular disease includes angina, stroke, or myocardial infarction.

Caregivers and noncaregivers were similar in general health status. Most baby boomers rated their health as excellent, very good or good, regardless of whether they were caregivers (75.8%) or noncaregivers (77.2%) (*P* = .09). However, baby boomer caregivers more often reported FMD (15.2%) than noncaregivers (10.3%) (*P* < .001). In addition, caregivers (63.4%) more often had at least 1 chronic health condition, compared with noncaregivers (57.3%) (*P* < .001). Caregivers more often had been diagnosed with arthritis (44.4% vs 37.0%; *P* < .001), current asthma (10.9% vs 9.1%; *P* = .002), and chronic obstructive pulmonary disease (11.2% vs 9.2%, *P* < .001) than noncaregivers. We found no difference in the prevalence of nonskin cancer, cardiovascular disease, and diabetes between baby boomer caregivers and noncaregivers.

Baby boomers most often cared for a parent (41.9%) (Table 2), although caring for another family member (25.3%), spouse or partner (17.3%), or a friend or neighbor (14.6%) also were common. More than half of caregivers (53.8%) had provided care for at least 2 years, and 28.6% were caregiving 20 hours or more per week. Most caregivers (79.4%) helped with the care recipient’s household tasks, and 50.5% assisted with their personal care. We found that women more often than men provided 20 hours or more of care per week and assisted with personal care.

**Table 2:** Characteristics of Baby Boomer Caregivers Overall and by Sex, Behavioral Risk Factor Surveillance System, 2015–2017^a^

Variable	All Baby Boomer Caregivers, Weighted % (Unweighted n = 26,617)	Female Baby Boomer Caregivers, Weighted % (Unweighted n = 17,327)	Male Baby Boomer Caregivers, Weighted % (Unweighted n = 9,290)	*P* Value^b^
Parent or parent-in-law	41.9	41.9	42.0	.90
Spouse or partner	17.3	16.8	18.0	.36
Other relative	25.3	27.4	22.0	<.001
Nonrelative	14.6	13.1	17.0	<.001
Missing	0.9	0.9	1.0	NA
**Caregiving ≥2 years**	53.8	53.4	54.4	.58
Missing	1.8	1.8	1.7	NA
**Caregiving ≥20 h per week**	28.6	31.6	23.9	<.001
Missing	5.9	6.3	5.3	NA
**Personal care**	50.5	53.9	45.1	<.001
Missing	1.1	1.0	1.1	NA
**Household tasks**	79.4	79.4	79.1	.79
Missing	0.9	0.9	1.6	NA

a 2015 states: Alabama, Florida, Idaho, Illinois, Indiana, Iowa, Kentucky, Louisiana, Maine, Mississippi, Nebraska, Pennsylvania, South Carolina, Virginia, West Virginia, Wisconsin, Wyoming; 2016 states/territories: Arizona, Arkansas, California, Colorado, Connecticut, District of Columbia, Georgia, Minnesota, Missouri, Montana, Nevada, North Dakota, Ohio, Puerto Rico, South Dakota, Tennessee, Texas; 2017 states: Alaska, Hawaii, Kansas, Maryland, Michigan, New Jersey, New Mexico, New York, Oklahoma, Oregon, Rhode Island, Utah.

b *P* value based on the χ^2^ test of weighted proportions comparing female and male caregivers.

We observed effect modification between caregiving and sex (*P* = .01) when we evaluated the relationship between caregiving and general health status (Table 3). Results were nearly identical (no changed point estimates or *P* values for caregiver status) when we included age or age^2^ in the models. Male caregivers had a higher prevalence of fair or poor health, compared with male noncaregivers of the same age (aPR = 1.17; 95% confidence interval [CI], 1.06–1.29; *P* = .001), although female caregivers and noncaregivers had the same prevalence of reporting fair or poor health (aPR = 0.98, *P* = .74). In models evaluating FMD and chronic health conditions, we observed no effect modification by sex. Caregivers were more likely to have FMD than their noncaregiving peers (aPR = 1.39; 95% CI, 1.26–1.53; *P* < .001), and caregivers were also more likely to have at least 1 chronic health condition, compared with noncaregivers (aPR = 1.10; 95% CI, 1.07–1.13; *P* < .001). Results were similar when we added educational attainment and race/ethnicity to the models. For models estimating fair or poor health among men and FMD, prevalence estimates indicated that caregivers were more likely to have negative health outcomes after accounting for differences in socioeconomic position.

**Table 3:** Prevalence Ratios for Associations Between Caregivers and General Health, Frequent Mental Distress, and Chronic Health Conditions Using Regression Models, Behavioral Risk Factor Surveillance System, 2015–2017^a^

Characteristic	Fair or Poor General Health aPR (95%CI) [*P* value^c^]	Frequent Mental Distress aPR (95%CI) [*P* value^c^]	≥1 Chronic Health Condition^b^ aPR (95%CI) [*P* value^c^]
**Women**			
**Caregiver status**			
Is a caregiver	0.98 (0.90–1.08) [.74]	—	—
Is not a caregiver	1 [Reference]	—	—
**Age, per year**	1.00 (0.99–1.01) [.85]	—	—
**Men**			
**Caregiver status**			
Is a caregiver	1.17 (1.06–1.29) [.001]	—	—
Is not a caregiver	1 [Reference]	—	—
**Age, per year**	1.01 (1.01–1.02) [<.001]	—	—
**Men and Women**			
**Caregiver status**			
Is a caregiver	—	1.39 (1.26–1.53) [<.001]	1.10 (1.07–1.13) [<.001]
Is not a caregiver	—	1 [Reference]	1 [Reference]
**Age, per year**	—	0.97 (0.97–0.98) [<.001]	1.03 (1.02–1.03) [<.001]
**Sex**			
Female	—	1.41 (1.28–1.55) [<.001]	1.07 (1.04–1.09) [<.001]
Male	—	1 [Reference]	1 [Reference]

Abbreviations: CI, confidence interval; aPR, adjusted prevalence ratio; — model not run.

a N = 109,268 from 2015 state data of Alabama, Florida, Idaho, Illinois, Indiana, Iowa, Kentucky, Louisiana, Maine, Mississippi, Nebraska, Pennsylvania, South Carolina, Virginia, West Virginia, Wisconsin, Wyoming; 2016 states and territories: Arizona, Arkansas, California, Colorado, Connecticut, District of Columbia, Georgia, Minnesota, Missouri, Montana, Nevada, North Dakota, Ohio, Puerto Rico, South Dakota, Tennessee, Texas; 2017 states: Alaska, Hawaii, Kansas, Maryland, Michigan, New Jersey, New Mexico, New York, Oklahoma, Oregon, Rhode Island, Utah.

b Chronic health conditions include arthritis, current asthma, nonskin cancer, cardiovascular disease, diabetes, and chronic obstructive pulmonary disease.

c *P* value based on survey weighted log-binomial regression models.

## Discussion

Using BRFSS data from 2015–2017, we examined the prevalence of baby boomer caregivers and compared their general health, mental health, and chronic health conditions with baby boomer noncaregivers. We found that caregiving is a common experience among baby boomers. One in 4 baby boomers is a caregiver, and although the generation represented about 23% of the US population (74 million) in 2016 (13), 38% of all caregivers are baby boomers. Typical baby boomer caregivers in our study were non-Hispanic white, college-educated, employed women, who were in good to excellent health and caring for their parents.

The general health of male baby boomer caregivers was poorer than male baby boomers who were not caregivers, although we found no significant difference in health status among female baby boomers, based on caregiving status. Given that female caregivers generally experience more stress than male caregivers (6,11), this result was somewhat surprising, because greater burden or stress is associated with negative health outcomes. One possible explanation for this observation is that male baby boomers might be more likely than their female caregiving counterparts to neglect their own health when providing care. Women tend to participate in more preventive care than men (14,15). However, we could not find any existing literature to indicate our hypothesis, and therefore it would need to be tested. Another explanation, given the cross-sectional nature of these data, is that in the baby boomer generation, men in poor health are more likely to assume a caregiving role while women are equally likely to become caregivers, whether their health is good or poor.

Across both sexes, caregivers more often had FMD. These patterns are well documented for caregivers of all ages, including other characteristics (ie, employed and unemployed) (16–18). Engaging caregivers in evidence-based training and support programs or increasing access to respite care or formal supports (eg, paid in-home assistance and caregiver duties) could reduce burden, which also could alleviate the FMD associated with caregiving (7).

We also found that caregivers were more likely than their noncaregiving counterparts to report chronic health conditions. Some studies suggest that caregiving can increase the risk for some chronic conditions, including heart disease, although some studies find no effect (7,19,20). Evidence also indicates that spouses and partners have similar perceived health and disability status, which might result from shared environments (21–23). Regardless, whether caregiving contributes to the development of chronic health conditions or reflects common risk factors, caregiving might affect chronic disease management. If caregivers are not able to attend to their own health (eg, attending medical appointments, taking medications as prescribed, being physically active, adhering to a healthy diet) because of the financial or time costs of caregiving, then their own health may be negatively affected. Caregivers might become unable to provide care and might even need a caregiver for themselves if negative conditions persist (7,9). Further research is warranted.

Our study showed that more than half of baby boomer caregivers provided care for longer than 2 years, and more than a one-quarter provided care for 20 hours or more per week. Previous studies have shown that the longer a person acts as a caregiver, including caregivers who are employed, the worse the caregiver’s health and mental health conditions become (4). Caregiving for aging parents for up to 4 years has resulted in significantly worse health among caregivers who are older baby boomers (16).

Previous studies showed that typical caregivers in all age groups (66%) are non-Hispanic white women (2). Our study confirms that finding, as most in our sample were non-Hispanic white (74%), employed (52%), and female (62%). However, previous studies indicated that the prevalence of caregiving is higher among black, Hispanic, and Asian families. These groups tend to care for aging family members because their cultures are family-oriented (24,25). In our study, the percentages of non-Hispanic black baby boomer caregivers (11.2%) and noncaregivers (10.5%) were similar (*P* = .25), but the percentages of caregivers were smaller than the percentages of noncaregivers among Hispanic (8.6% vs 12.6%; *P* < .001) and Asian or Pacific Islander (1.1% vs 4.1%; *P* < .001) baby boomers. The results of our study support the idea that caregiving responsibilities may be shared among family members, as many African American, Hispanic, and Asian families do (26,27). Although some Hispanic and Asian baby boomers are providing assistance to aging persons, they might not consider themselves as caregivers because caring for their parents is perceived as normal and expected in their cultures. Growing up, some of these caregivers observed their mothers and fathers caring for their parents; therefore, caregiving is not new to them (27).

More studies on baby boomers in general, and baby boomers as caregivers in particular, are warranted. As the number of older adults increases, more caregivers will be needed to meet their physical, mental, and cognitive needs. We saw in our study that baby boomers are one large cohort that provides informal care for people with long-term illnesses, disabilities, or chronic health conditions, and this care frequently involves help with personal care and household tasks. Without the help that baby boomers provide, many of those who need assistance might have unmet needs or require formal or institutional care. However, the baby boomer generation of caregivers is also aging and might need their own caregivers. In 2018, there were 7 potential family caregivers per 1 adult. By 2030, when all baby boomers will be aged 65 years or older, there will be only 4 potential family caregivers per 1 adult, increasing the burden on the caregiver workforce (10). The number of caregivers might not be sufficient to provide care for baby boomers when they need care. Strengthening that workforce is a logical and plausible recommendation.

The population aged 65 or older is projected to increase from 52 million in 2018 to 71 million in 2030, when the last cohort of baby boomers will turn 65. Therefore, it is imperative to have concrete plans to support this large cohort of aging baby boomers. CDC’s Alzheimer’s Disease and Healthy Aging Program and the Alzheimer’s Association developed Supporting Caregivers: A Healthy Brain Initiative Issue Map, which is framed on the basis of essential public health services and identifies 17 public health actions to support caregivers (28). The Issue Map is an example of a strategy for supporting baby boomer caregivers. Although it was designed for people providing care for someone with Alzheimer’s disease or dementia, the Issue Map might be used as a template to support baby boomer caregivers.

Limitations of our study include its cross-sectional nature and the lack of detail in some characteristics of caregivers. Because we only knew the caregivers’ health status at the time of the survey, it is possible that the physical burden of caregiving did not negatively affect their physical health status. Instead, chronic stress from caregiving might have negatively contributed to their poor health (29). Given previous research on the negative health effects of caregiving, caregiving most likely did contribute to the negative health affects observed (20,29). Longitudinal studies would help to clarify the relationships observed. State-level variation in services, supports, and access to quality care for older adults or caregivers might result in lower prevalence ratios for FMD, for example, but we did not investigate this variation. Another limitation is that BRFSS data are self-reported, and responses are subject to biases, such as social desirability bias. Finally, because we did not have data about chronic disease self-management, we could not assess whether the level of chronic disease self-management for caregivers differed from that of noncaregivers. Despite these limitations, our study contributes to the literature because we used a large, representative sample covering 44 states, included racial/ethnic minority populations who are often underrepresented in research, and used validated measures.

Our study enhances knowledge on the prevalence of baby boomer caregivers and their physical and mental health status. As people age, more baby boomers might serve as caregivers. To enable their performance in this role as long as possible, public health efforts are needed to support the caregiver role and enhance their health. This support might include improving care for older adults and providing supports outside of family systems. For example, a community implementation of the evidence-based Resources for Enhancing Alzheimer’s Caregiver Health (REACH) II program (30) involved a 6-month multicomponent, psychosocial intervention (6 face-to-face sessions, 6 telephone sessions, telephone support groups). Caregivers reported significant decreases in “depression, burden, and bother” by care recipients’ memory problems at 6 and 12 months, showing the successful implementation of the program through a partnership between developers and community partners. About half of baby boomer caregivers were employed in our study, suggesting that caregiving policies for older adults in the workplace could be modified to ease the burden of working while caregiving. To support caregivers broadly, other actions suggested in the Healthy Brain Initiative Issue Map should be considered.
